# Enhanced Strong-Field Ionization and Fragmentation of Methanol Using Noncommensurate Fields

**DOI:** 10.1021/acs.jpca.4c05584

**Published:** 2024-10-03

**Authors:** Eladio Prieto, Rituparna Das, Naga Krishnakanth Katturi, Jacob Stamm, Jesse Sandhu, Sung Kwon, Matthew Minasian, Marcos Dantus

**Affiliations:** † Department of Chemistry, Michigan State University, East Lansing, Michigan 48824, United States; ‡ Department of Physics and Astronomy, Michigan State University, East Lansing, Michigan 48824, United States; § Department of Electric and Computer Engineering, Michigan State University, East Lansing, Michigan 48824, United States

## Abstract

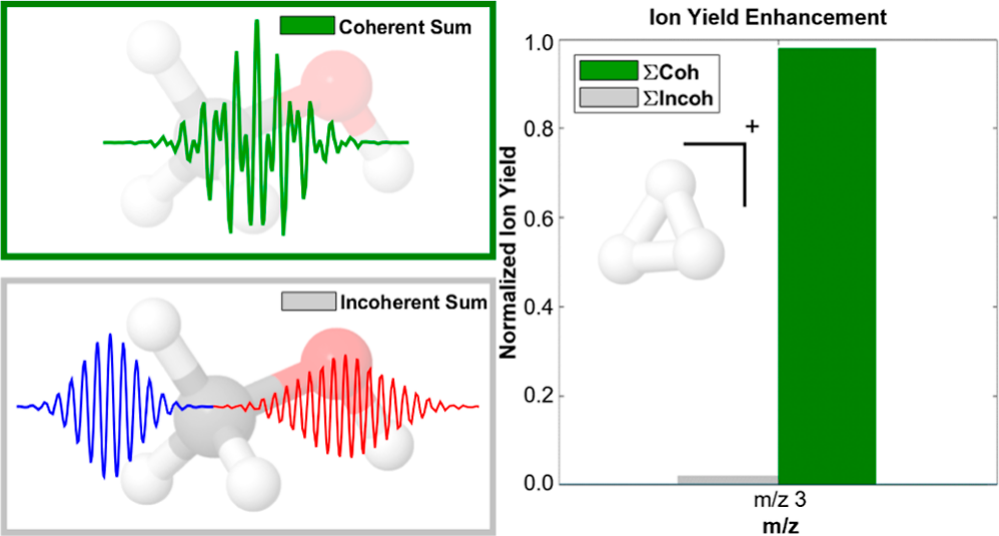

Electron-initiated chemistry with chemically relevant electron energies (10–200 eV) is at the heart of several high-energy processes and phenomena. To probe these dissociation and fragmentation reactions with femtosecond resolution requires the use of femtosecond lasers to induce ionization of the polyatomic molecules via electron rescattering. Here, we combine noncommensurate fields with intensity-difference spectra using methanol as a model system. Experimentally, we find orders of magnitude enhancement in several product ions of methanol when comparing coherent vs incoherent combinations of noncommensurate fields. This approach not only mitigates multiphoton ionization and multicycle effects during ionization but also enhances tunnel ionization and electron rescattering energy.

## Introduction

Electron-initiated chemical reactions (EICRs) are crucial for both fundamental and applied research, finding applications in diverse fields, such as mass spectrometry, photolithography, combustion, plasma processing, atmospheric chemistry, ionizing-radiation medicine, and astrochemistry. Hence, there is a pressing need to move beyond a thermodynamic and statistical description to a molecular-level time-resolved understanding of EICRs. Achieving femtosecond time resolution and energy resolution simultaneously has proven challenging for chemically relevant electron energies (10–200 eV), where the electron scattering cross-section is maximized. Fortunately, electron rescattering following tunnel ionization via irradiation with femtosecond near-infrared pulses has emerged as a promising approach for achieving femtosecond and even attosecond time resolution for chemically relevant electron atom/molecule interactions.^1−3^ Given that the molecules themselves are the source of the electron that causes their own ionization, this approach allows for the time-resolved measurement of EICRs.^4,5^ Unfortunately, the ionization process induced by a femtosecond laser pulse may involve unwanted effects (such as multiphoton ionization) depending on its energy, pulse duration, and wavelength.^6,7^ These unwanted effects are less likely to induce electron rescattering, partially due to their relationship between the ionization probability and the laser phase. To address these challenges in EICRs, we propose the interaction of a pair of femtosecond laser pulses with noncommensurate (NC) wavelengths to exploit the relatively phase-insensitive constructive interference in a minority of the optical cycles.^8−10^ Two wavelengths are called “noncommensurate” if their respective frequencies are not integer multiples of each other. Furthermore, these NC fields create sparse constructive inference optical cycles in the temporal intensity profile of the pulse. Additionally, we suggest employing intensity-difference spectra (IDS) to mitigate contributions from the leading and trailing wings of the laser pulses, as well as the lower energy regions of the Gaussian radial distribution of pulse energy, where multiphoton ionization is prevalent.^11−13^ Notably, for several pathways, we observe an order of magnitude discrimination when comparing ion yields obtained following excitation by the coherent compared to the incoherent sum of the two fields. This paper presents the theory and implementation of this approach, which we plan to utilize in future time-resolved studies.

The ionization of atoms and molecules using commensurate and NC fields has been an active field of research for decades. When combining the fundamental and second harmonic fields, the above-threshold ionization rate was enhanced and found to be phase-sensitive.^14^ This inspired subsequent studies^15,16^ to investigate the effects of relative phase-dependent forward–backward field asymmetry and the relative polarization^17,18^ on ionization yield,^19,20^ the photoelectron momentum distribution,^21−23^ the directional ejection of strong-field ionized fragments,^24−26^ and the control (enhancement) of the tunneling ionization and high-order harmonic generation.^27−30^ The addition of NC wavelengths, e.g., 1290 and 780 nm, results in enhanced yield of midharmonics and higher cutoff energy, as reported by Siegel et al.,^31^ which was followed by Takahashi et al.^32^ and Lan et al.^33^ generating isolated attosecond pulses with a continuum high-order harmonic spectra^8^ using multicycle pulses with carrier-envelope phase (CEP) insensitivity by optimizing the wavelength of the assisting field. Due to the constructive and destructive interference between the NC fields, the resultant field consists of well-separated optical cycles with altered tunneling ionization rates and subsequent subcycle electron dynamics.^9,10^ While NC fields have been shown to be useful by themselves, ideal energy resolution requires the elimination of unwanted field effects from the femtosecond pulses. This is why we combine the advantages of using NC fields with the IDS method,^11−13^ which accounts for the focal volume effect arising from the molecular ensemble interacting with a laser pulse, which is Gaussian both spatially and temporally. This combination enables us to distinguish between fragmentation pathways arising from tunnel ionization and multiphoton ionization.

We focus on the dissociative ionization of methanol as a model polyatomic system, which has been extensively studied by electron impact with energies ranging from 10 to 500 eV.^34−39^ This molecule is used as a model for molecules involving O, C, and H in the interstellar medium,^40^ and it has been fundamental in studying the formation and reaction of H_3_^+^.^41−45^ Here, we apply femtosecond NC fields and IDS to the strong-field ionization of methanol in order to study the fragmentation in isolated regions of single-electron tunnel rescattering without contributions from other mechanisms, such as multiphoton ionization.

## Theoretical Concept

We now investigate the dependence of the strong-field ionization process on the temporal characteristics of the NC fields. Mathematically, the NC field can be represented as
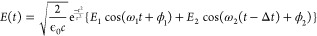
1where *E*(*t*) is the resultant NC field and *c* and ϵ_0_ are fundamental constants. τ is a constant related to the pulse duration, *E*_1_ and *E*_2_ are the electric field amplitudes of each pulse, and ω’s and ϕ’s represent the central angular frequency and carrier-envelope phase for each pulse, respectively. Δ*t* is the time delay between pulses. With this description of the NC fields, we can begin predicting the regime of ionization that is expected to be dominant at different points in the field. This is commonly applied to atoms by using the Keldysh parameter
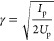
2

This parameter helps delineate between the two limiting cases of strong field ionization: the multiphoton regime (γ ≫ 1) and the tunneling ionization regime (γ ≪ 1). It is this second regime that we wish to isolate to study electron-initiated chemistry. Once this regime is isolated, one can compute the energy incident on the molecule via the rescattered electron using the equation for the ponderomotive energy
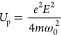
3where the rescattered electron can achieve at maximum a constant multiple of this energy (3.17 *U*_p_) for a monochromatic field.^1^ Note that to achieve the maximal electron kinetic energies, one wishes to decrease the frequency of the ionizing field or combine multiple frequencies as is done here to raise this kinetic energy ceiling.

**Figure 1:**
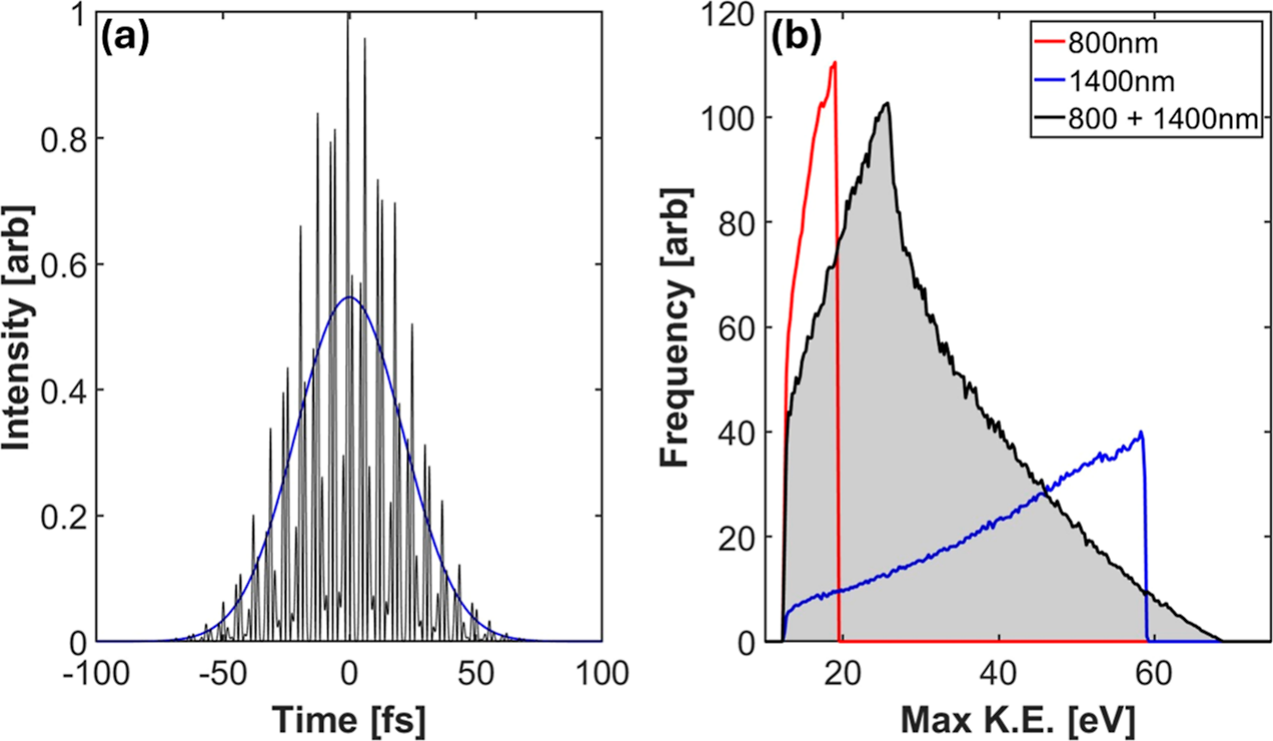
Calculations of the NC fields at 800 and 1400 nm and their resulting electron kinetic energy spectrum upon rescattering. Panel (a) shows the temporal intensity profiles of the combined 800 nm (2.5 × 10^13^ W cm^–2^) and 1400 nm (7.5 × 10^13^ W cm^–2^) fields (black line) with the envelope of a single-color field at the total intensity of 1 × 10^14^ W cm^–2^. The NC field has a field maximum of 3.7 V/Å, while the single-color fields have a maximum of 2.8 V/Å. Panel (b) shows the calculated electron kinetic energy distribution upon rescattering for the NC field and for the single-color case for 800 and 1400 nm. Details about how this panel was calculated are discussed in the Methods section. A version of panel (a) with different relative phases and (b) utilizing the IDS method is shown in Figures S1 and S2.

The single color (800 and 1400 nm) versus combined intensity profiles are shown in Figure 1a. First, we observe that for the same total peak intensity, the 25%/75% ratio of 800/1400 nm shows significantly larger peaks in the temporal intensity stemming from optical cycles coinciding in phase leading to constructive interference within the pulse envelope, from now on referred to as spikes. Second, the intense spikes are spaced further away (∼6 fs) from one another in comparison to a strong monochromatic field, which in the case of 800 nm has a field maximum every 1.33 fs (for the single-color fields, we only plot the intensity envelope). In the resulting electron kinetic energy spectra following rescattering, shown for the single-color cases and the NC case in Figure 1b, we observe sharp cutoffs for the single-color fields at the theoretically predicted value (3.17 *U*_p_). Despite having the same total intensity, however, the combined NC field extended this cutoff beyond the 1400 nm ponderomotive limit to yield even higher electron kinetic energies. The λ^2^ dependence of the ponderomotive energy (eq 3) causes the optimal ratio for enhancing the electron’s rescattering energy to be biased toward the 1400 nm. We find that the 25%/75% intensity ratio for the wavelengths used is optimal for maximizing the electron kinetic energy. Under these NC field conditions, one expects significantly higher internal energy to be deposited into the molecule by the rescattering electron compared to ionization by either single-color fields separately, even when the total laser intensity is matched. In addition, ionizing the molecule by rescattering such energetic electrons is expected to give rise to fragments having a higher appearance energy (AE). This is demonstrated experimentally in the following sections for gas-phase methanol.

As mentioned above, we employed the IDS method to mitigate the contributions of the lower-intensity regions of the Gaussian laser pulse to the ionization dynamics initiated by the NC field. Wiese et al. computed the intensity distribution of the pulse by taking both the Gaussian spatial and temporal profiles into account.^13^ In this 3D configuration, it was shown that the differential volume occupied by the isointensity shell d*I* around *I* is given by
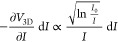
4which indicates the weight of intensity in the range of 0 to *I*_0_ on the interaction of the pulse with the molecular beam. It must be noted that while computing the volume function *V*_3D_, it is assumed that the radial intensity distribution is independent of the direction of laser beam propagation *z* since *D* < 2*z*_R_, where *D* and *z*_R_ are the molecular beam diameter and the Rayleigh length, respectively. In the present experiments, this interaction region is limited by a 1 mm slit in the extractor plate.

**Figure 2:**
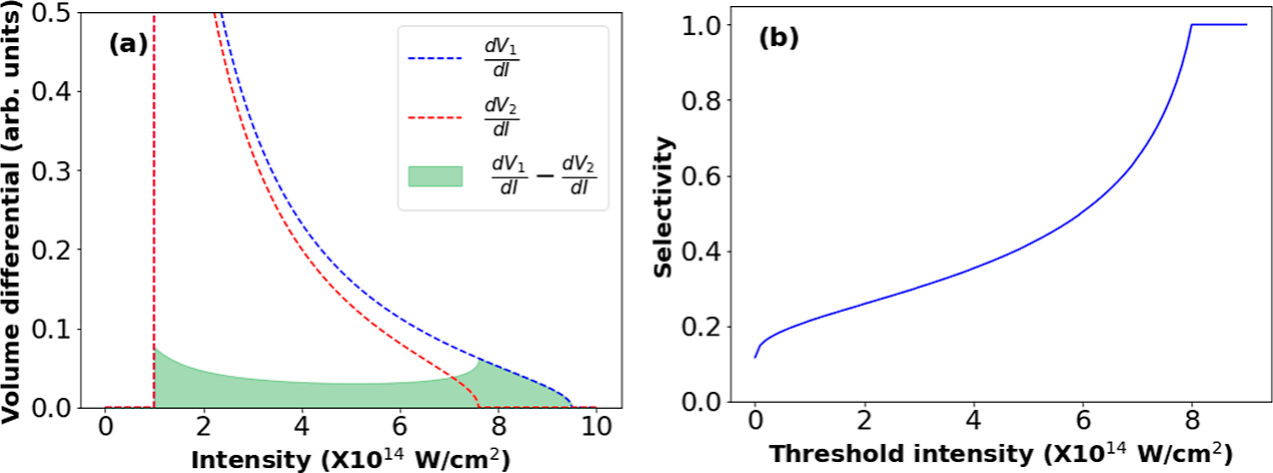
Calculations illustrating the IDS method. Panel (a) plots the volume differential for peak intensity *I*_0_ (blue) and 0.8*I*_0_ (red). The green region corresponds to the difference between the two volume differentials. Panel (b) plots the ion appearance selectivity as a function of increasing threshold intensity. The *y*-axis represents the ratio of the areas under the difference volume differential (Δ∂*V*/∂*I*) obtained from species with a high threshold (8 × 10^14^ W/cm^2^) and those with a much lower threshold.

The IDS method is conceptually discussed here. Figure 2a shows the volume differential for the two peak intensities *I*_0_ (blue) and 0.8*I*_0_ (red). Assuming that the contribution to the ion yield from regions of the pulse with intensities less than 10^14^ W/cm^2^ is extremely low, we have considered the threshold ionization intensity to be 10^14^ W/cm^2^ in this case, meaning that this intensity is the cutoff for when ionization can begin to occur for the molecule of interest. The green-shaded region refers to the difference between the volume differentials for the two values of peak intensity. The figure shows that the contribution to Δ∂*V*/∂*I* by intensities smaller than 0.8*I*_0_ is highly suppressed. In Figure 2b, we plot the selectivity of the IDS method in the appearance of ions with increasing AE. The threshold intensity representing the minimum laser intensity at which an ion species appears is plotted along the *x*-axis. This plot indicates that for ion species having higher threshold intensities, the selectivity of the IDS method is high.

## Methods

In brief, the experimental setup is described below. The two-color field comprising noncommensurate frequencies was obtained by overlapping parallel polarized pulses having central wavelengths of 800 and 1400 nm. A Ti:sapphire laser (ASTRELLA, Coherent Inc.) producing 800 nm, 5 mJ, 1 kHz pulses was split 50/50 using a beam splitter, a part of which was sent into an optical parametric amplifier (TOPAS Prime, Light Conversion) to produce tunable laser pulses from 260 to 2600 nm. The 1400 nm beam was expanded using a pair of lenses to ensure that both beams had the same focal length and Rayleigh lengths when collinearly combined and focused by a single lens. The 1400 and 800 nm pulses were measured to have a pulse duration of 53 and 168 fs, respectively. The beams were collinearly combined and focused into a Wiley–McLaren time-of-flight (TOF) mass spectrometer. The intensities of the pulses were controlled by using variable attenuators. The two-color pulses were focused in the ionization region of the TOF spectrometer with an achromatic doublet lens (focal length of 200 mm).

The average intensities maintained during the experiment were 2.1 × 10^14^ W/cm^2^ for 800 nm and 1.1 × 10^14^ W/cm^2^ for 1400 nm. These intensities were determined in situ by measuring the ratio of Ar^2+^/Ar^+^.^46^ The Keldysh parameter for the strong-field ionization of methanol (ionization potential of 10.85 eV) was found to be 0.38 and 0.157, respectively. This indicates that the ionization by the two-color pulses occurs predominately via tunnel ionization. Furthermore, the NC mass spectrum of methanol is in good agreement with the experimental tunnel ionization mass spectrum of methanol in Rajgara et al., where an electron rescattering mechanism was concluded.^47^ Dry methanol was effused into the TOF chamber by using a needle valve. The baseline chamber pressure was 9 × 10^–8^ Torr, while the pressure was maintained at 2 × 10^–6^ Torr, corresponding to a density of 7 × 10^10^ molecules/cm^3^ throughout the experiment. The ion TOF signals were detected using a microchannel plate (MCP, RM Jordan) detector and digitized using an oscilloscope (LeCroy WaveRunner 610Zi, 1 GHz). Each scan was composed of 300 laser shots and was repeated 64 times.

The simulations in Figure 1b result from a trajectory-based classical mechanics simulation of the free electron in the optical field. For each trajectory, the electron is born at a random point within the NC field (where the field is given a random phase) and evolved according to classical mechanics under the action of the electric field of the pulse. If the electron returns within a certain radius of the nucleus (a radius given by the scattering cross-section of the electron at its instantaneous kinetic energy), the trajectory is labeled with that kinetic energy. This result is then scaled according to the ADK probability that the electron was birthed at a starting point given the pulse’s electric field at that time. This process was done for 5000 random NC phases, and each phase was run with 5000 random electron birth points. The histogram of the resulting rescattering energies is shown in Figure 1b. Both NC fields were parallel-polarized, meaning that no elaborate electron orbits occurred, and the combined fields only acted to increase the electron kinetic energy ceiling.

## Results and Discussion

**Figure 3:**
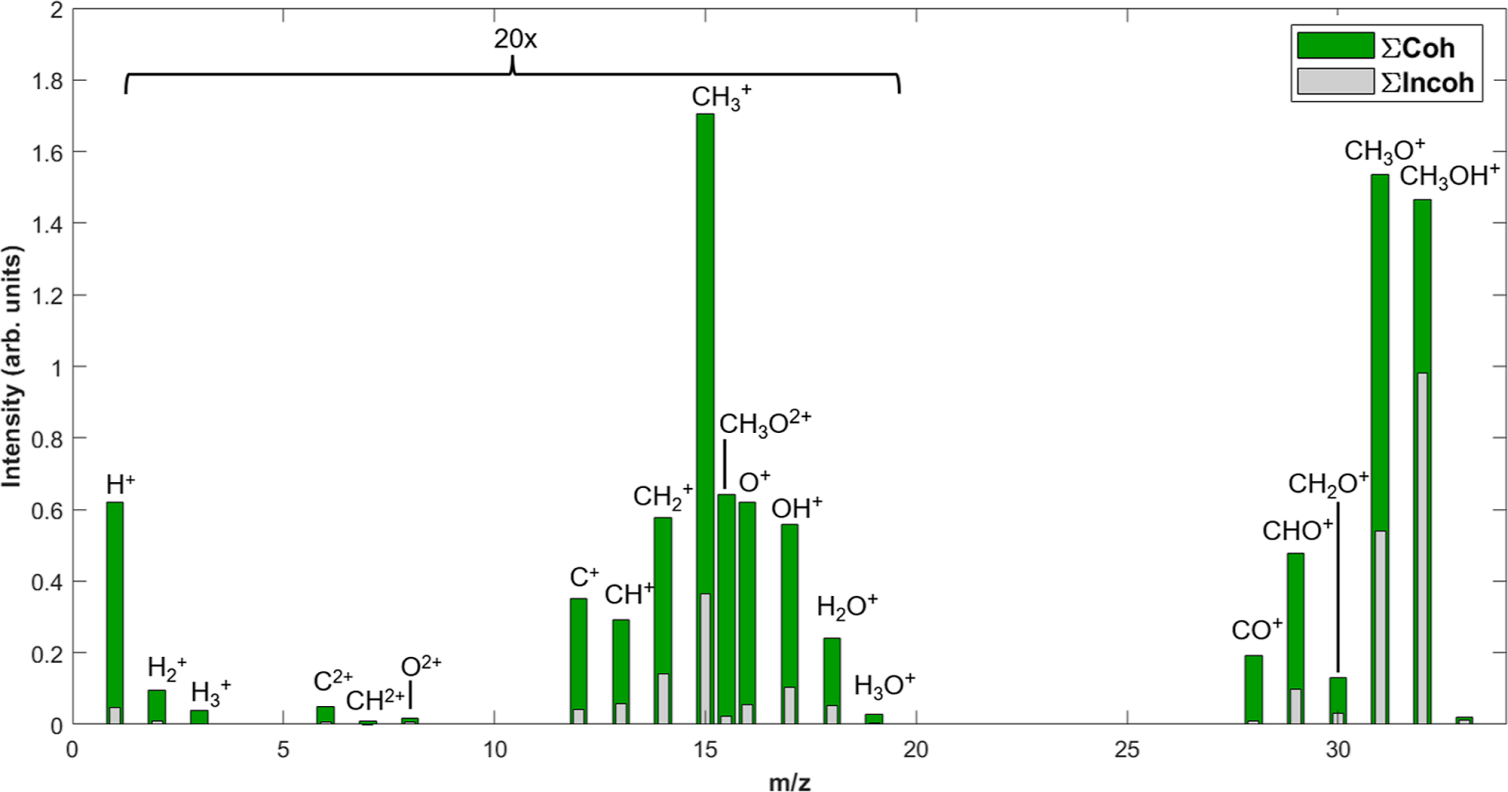
IDS of methanol with both pulses temporally overlapped (green, coherent sum) and sum of individual pulses (gray, incoherent sum). For the coherent sum, 2.1 × 10^14^ W/cm^2^ 800 nm, 1.1 × 10^14^ W/cm^2^ 1400 nm and 1.6 × 10^14^ W/cm^2^ 800 nm, 8.7 × 10^13^ W/cm^2^ 1400 nm were used. For the incoherent sum, the same intensities were used individually and the resulting spectra summed. Additional results for coherent and single-color fields that produce equivalent total ion yields are shown in Figure S3.

We ionized methanol with the NC laser fields overlapped in time using two different sets of intensities (2.1 × 10^14^ W/cm^2^ 800 nm/1.1 × 10^14^ W/cm^2^ 1400 nm and 1.6 × 10^14^ W/cm^2^ 800 nm/8.7 × 10^13^ W/cm^2^ 1400 nm). The resulting IDS obtained by the difference between these two conditions is shown in Figure 3. The spectrum is displayed with the coherent sum (∑Coh) represented in green, indicating the combined effect of the overlapping fields. We compare this result with the IDS obtained from the incoherent sum (∑Incoh) sum of the individual wavelengths at the same powers represented in gray. The analysis reveals an enhanced ion yield from the coherent sum for all ions, contrasting with the lower ion yield observed for the incoherent sum. This enhancement underscores the significant internal energy imparted to the molecule upon overlapping the two pulses. For the incoherent sum, the spectrum primarily contains the molecular ion (CH_3_OH^+^) and CH_3_O^+^, along with a very low yield of CH_2_O^+^, CHO^+^, and CH_3_^+^ (note the 20× region in Figure 3), implying a relatively low internal energy imparted following ionization. From these results, we may infer that the intensities of the individual single-color fields are sufficient to primarily singly ionize the molecules and fragmentation is insignificant. However, on temporally overlapping the two pulses, causing spikes in the NC electric field, we not only observe a significant enhancement in the yields of the ions observed in the previous case but also the appearance of smaller fragments mainly originating from dication species such as H_*n*_^+^ (*n* = 1–3) and CH_*n*_^+^ (*n* = 0–3). Despite this, the primary initial charge state of the methanol cation (as determined by the ion yields and identities) in both cases is singly ionized. Ionization with NC fields simply increases the mean internal energy of the methanol molecule by double ionization via electron rescattering. In fact, the enhancement measured is dependent on the AE of the ion, as shown in Table 1. This could be explained by the NC field confining energy in selective optical cycles formed by the constructive interference of the fields, causing greater energy to be given to the rescattering electron. This enhances the ion yield by more than an order of magnitude for high-AE fragments. The appearance energies of several fragments of methanol are shown in Table 1.

**Table 1:** Appearance Energies and Degree of Enhancement of Combined Two-Color Fields for Several Key Product Ions from the Ionization of Methanol

product ion	*m*/*z*	appearance energy (eV)	other products	enhancement
CH_3_OH^+^	32	10.85^a^		1.5×
CH_3_O^+^	31	11.67^a^	H	2.8×
CH_2_O^+^	30	10.9^a^	H_2_	4.2×
CHO^+^	29	13.6^a^	H_2_ + H	4.8×
CO^+^	28	13.7^a^	2H_2_	19.4×
CH_3_OH^2+^/O^+^	16	32.4^b^, 30.8^c^/12.0^a^		4.5×
CH_3_O^2+^	15.5	33.7^b^		45.5×
CH_3_^+^	15	13.82^a^	OH	4.7×
CH_2_^+^	14	14.05^a^	H_2_O	4.3×
CH^+^	13	22.31^a^		5.6×
C^+^	12	24.4^d^	2H_2_ + O	10.1×
C^2+^	6	47.9^d^		>60×
H_3_^+^	3	31^c^		21.4×
H_2_^+^	2	26.5^e^	OH + C + H	16.2×
H^+^	1	21.5^e^	H_2_O + CH	15.4×

a Data obtained from NIST.^48^

b Douglas and Price.^49^

c Eland and Treves-Brown.^50^

d Atomic ionization energy.^51^

e Burton et al.^52^

We can relate the AE to the degree of enhancement as follows: according to the previous discussion, the main difference between ∑Coh and ∑Incoh is the increase in the rescattering energy of the electron. Therefore, it is expected that ions requiring more energy for their creation show the greatest difference. Indeed, ions with a higher AE exhibit the largest enhancement in energetic order. Specifically, C^+^ < H^+^ < H_2_^+^ < H_3_^+^ < CH_3_O^2+^ < C^2+^ (see Table 1) align with the theoretical predictions. In the case of C^2+^, the ∑Incoh mass spectrum showed no signal at this *m*/*z*; therefore, the number given is based on the experimental noise floor. On the other hand, ions with low AE (with the exception of CO^+^) show poor enhancement. One possible explanation for the greater-than-expected enhancement observed for CO^+^ is that this ion could also be created through a high-energy mechanism, such as the fragmentation of doubly charged methanol (CH_3_OH^2+^), as shown in the following reaction.^49^
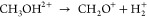

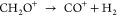


**Figure 4:**
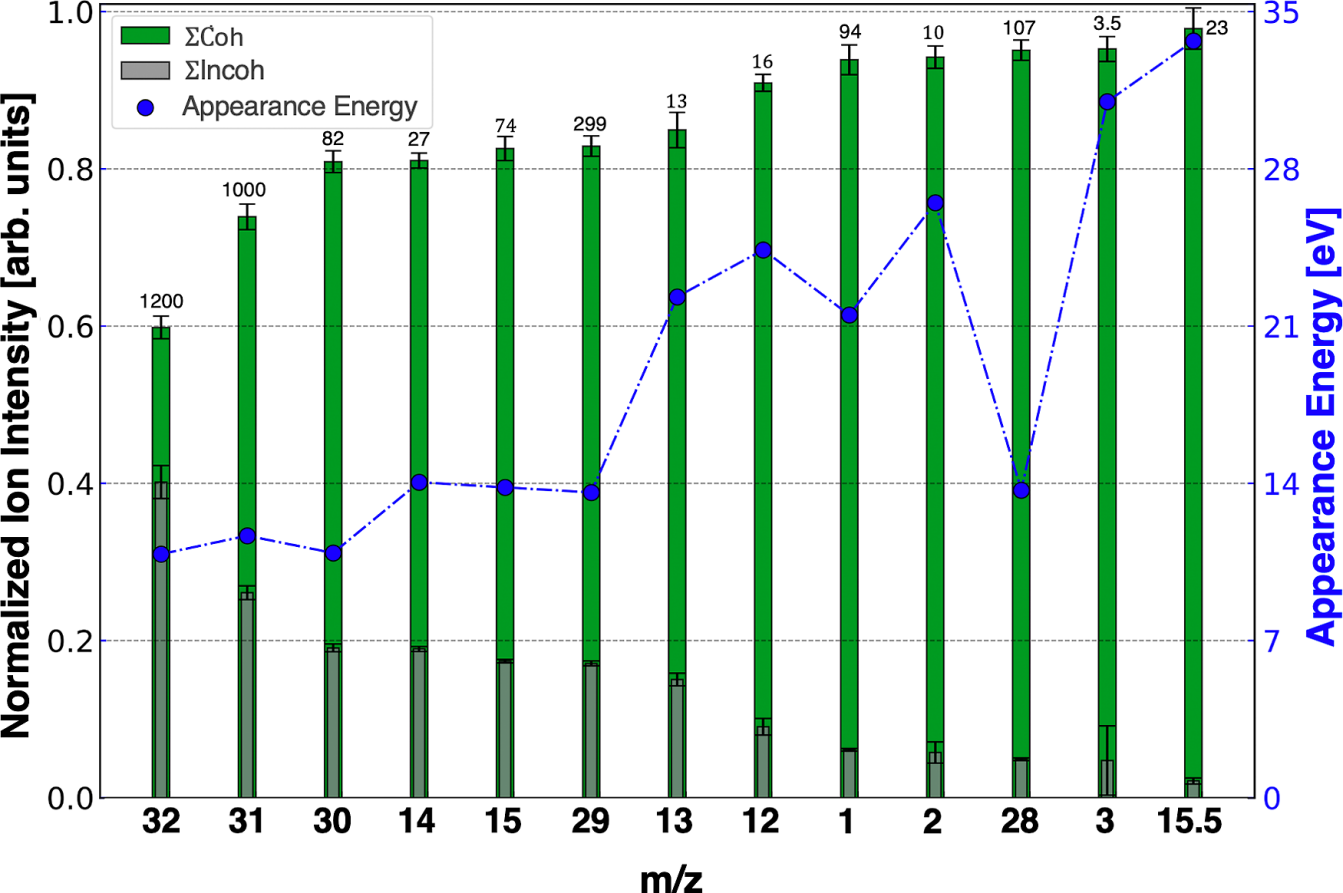
Bar graph of selected ions from IDS that have been normalized such that the incoherent sum (gray) plus the coherent sum (green) equals one. The number on top of each bar indicates the factor by which the raw ion yield has been divided to normalize it. The error bars display ±1 standard deviation of the ion yield from the 64 scans. The blue circles in each bar, connected by a dashed line, correspond to the appearance energy of each fragment ion.

Considering that the threshold of the first fragmentation requires approximately 31.5 eV^50^ and that an excess of the internal energy of CH_2_O^+^ is necessary to produce CO^+^, it is reasonable to observe the enhancement of this ion. We expect high-energy mechanisms to explain other departures from the trend in Figure 4, such as H^+^. By normalizing the experimental results such that the combined yield of coherent and incoherent sums equals one, we achieve a clearer representation of the enhancement, as illustrated in Figure 4. Upon arranging the results from minimum to maximum enhancement, a correlation emerges between the enhancement and the appearance energy of each fragment ion. Notably, CO^+^ stands as an outlier, as previously noted. This correlation aligns with insights from the analysis depicted in Figure 2b, where the IDS method’s selectivity correlates with the threshold intensity needed to detect a specific ion.

## Conclusions

The effect of combining noncommensurate laser field ionization with the IDS method to study the dissociative ionization of polyatomic molecules is demonstrated for methanol. We observed over an order of magnitude increase in specific ionization pathways when using NC fields when compared to the incoherent sum of fields, an enhancement correlated with their respective appearance energies. This effect is attributed to sparse instances of constructive interference between the noncommensurate optical cycles, leading to a greater kinetic energy gain of the rescattered electron. These findings confirm what had been observed for high harmonic generation, namely, the generation of electrons with higher kinetic energy achieved by the coherent sum of NC fields, determined by the presence of high appearance-energy fragments. These results allow for the study of chemistry induced by electrons with chemically relevant energies, in particular, when the energy of the individual fields is insufficient. Additionally, the use of NC fields restricts ionization to individual instances in which constructive interference occurs between the two optical fields. The combination of NC fields with IDS minimizes the contribution from multiphoton ionization. Future experiments in our laboratory will explore other pulse parameters, such as different wavelengths, polarizations, and pulse durations, to control electron-initiated reactions.

## Supporting Information Available

Additional experimental details and theoretical calculations about NC models (PDF)
