# Supplementary material for: Enhanced Strong-Field Ionization and Fragmentation of Methanol Using Noncommensurate Fields
Source: J Phys Chem A. 2024 Oct 3;128(41):9099–106. doi: 10.1021/acs.jpca.4c05584 (PMC11492292; doi:10.1021/acs.jpca.4c05584)
Supplement: Supplementary file 1 — jp4c05584_si_001.pdf [file jp4c05584_si_001.pdf]

Supporting Information: Enhanced Strong-Field Ionization and Fragmentation of Methanol Using Non-Commensurate Fields

Eladio Prieto,[†] Rituparna Das,[†] Naga Krishnakanth Katturi,[†] Jacob Stamm,[†] Jesse Sandhu,[†] Sung Kwon,[†] Matthew Minasian,[†] and Marcos Dantus*,[†,‡,¶]

†*Department of Chemistry, Michigan State University, 48824 East Lansing, MI, United States*

‡*Department of Physics and Astronomy, Michigan State University, 48824 East Lansing, MI, United States*

¶*Department of Electric and Computer Engineering, Michigan State University, 48824 East Lansing, MI, United States*

E-mail: dantus@chemistry.msu.edu

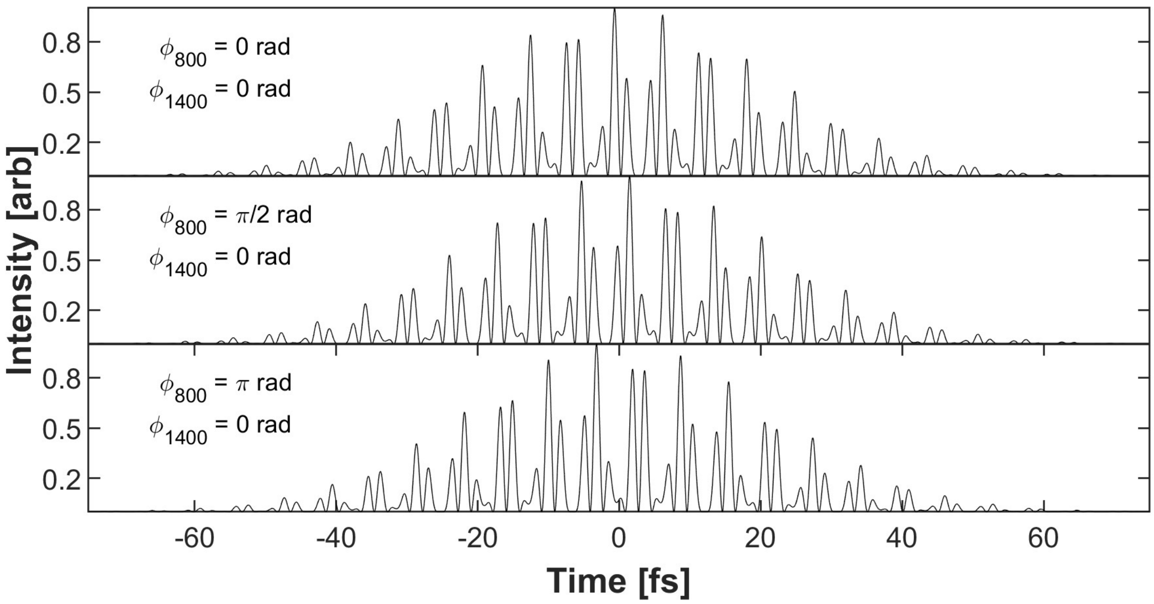

Figure S1: Three NC intensity profiles for different phase delays between the 800 and 1400 nm fields. Note how separated spikes appear in all three cases.

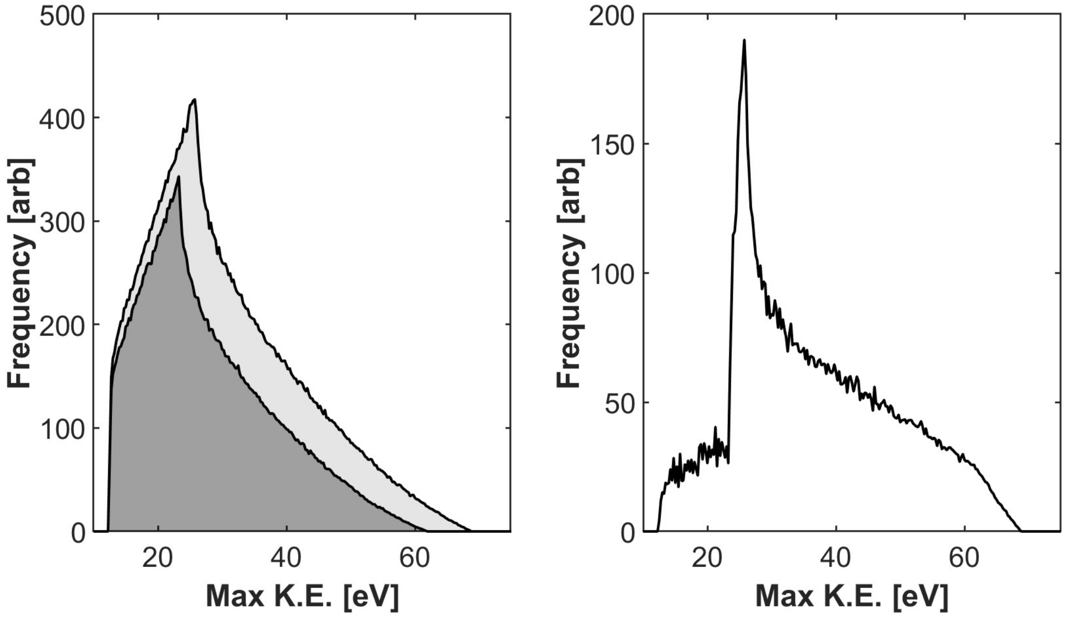

Figure S2: Intensity-difference spectra (IDS) methodology applied to Figure 1(b) of the main manuscript. The left panel shows the calculated electron kinetic energy distribution upon rescattering for the NC fields involving the combination of 800 nm (2.5×10^{13} W cm^{-2}) and 1400 nm (7.5×10^{13} W cm^{-2}) fields (lighter shaded curve) and 80% the intensity of both wavelengths (darker shaded curve). The right panel shows the difference between the electron kinetic energy distributions in the left panel, showing that the lower kinetic energy components are significantly reduced.

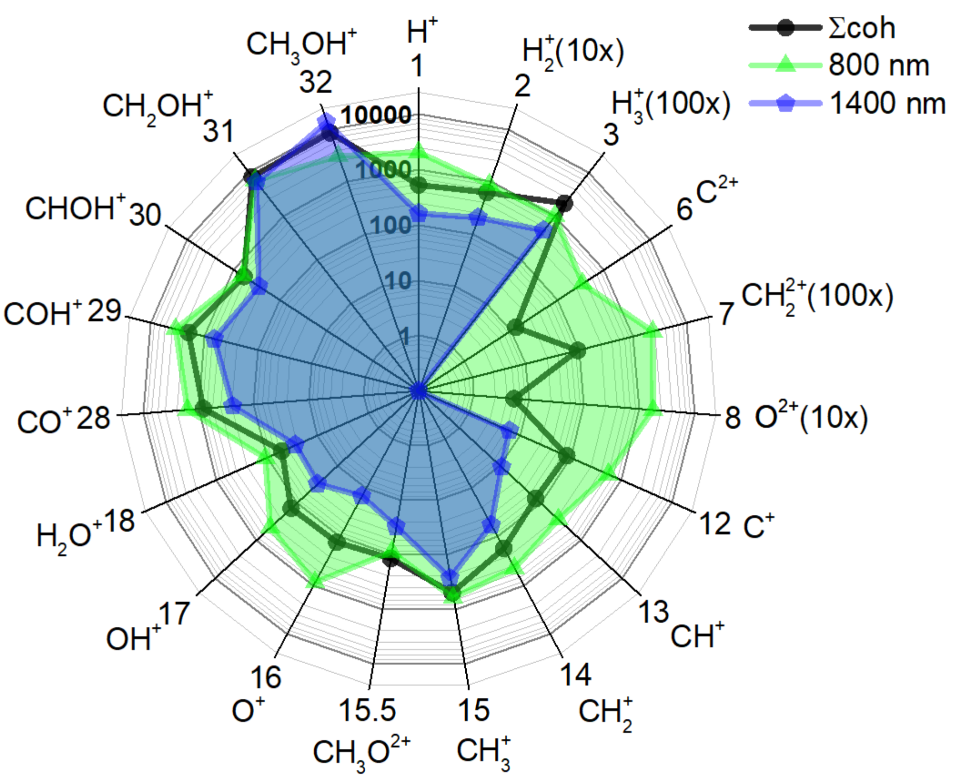

Figure S3: Comparison of enhancement using NC field and individual beams with the same total ion yield. NC field (Σcoh) - 1400 nm at 3.9 × 10^{14} W cm^{-2} and 800 nm at 9.1 × 10^{13} W cm^{-2}, 800 nm - 2.6 × 10^{14} W cm^{-2}, 1400 nm - 6.7 × 10^{14} W cm^{-2}.
